# Perventricular closure of muscular ventricular septal defects: How do I do it?

**DOI:** 10.4103/0974-2069.41052

**Published:** 2008

**Authors:** Karim A. Diab, Qi-Ling Cao, Ziyad M. Hijazi

**Affiliations:** Heart and Lung Institute, Scott and Laura Eller Congenital Heart Center, St. Joseph's Hospital and Medical Center, Phoenix, AZ, USA; 1 Rush Center for Congenital and Structural Heart Disease, Rush University Medical Center, Chicago, IL, USA

**Keywords:** Hybrid procedure, congenital heart defects, device closure

## INTRODUCTION

Muscular ventricular septal defects (mVSDs) account for approximately 10-15% of all VSDs.[1] These defects are entirely located in the muscular portion of the ventricular septum, and can be further classified in order of their frequency into apical, mid-septal, anterior, and posterior defects. Indications for closure include large defects resulting in left ventricular volume overload, prevention of pulmonary hypertension, and the development of endocarditis.[2] In addition, when associated with other complex congenital heart defects such as double-outlet right ventricle (RV) and transposition of the great arteries, multiple mVSDs or Swiss cheese VSDs become a significant risk factor for early mortality.[3,4] Despite various approaches, surgical repair of mVSDs continues to be associated with significant long-term morbidity and mortality.[5–8] Surgical right atrial or right ventricular approaches provide suboptimal visualization of the defects due to heavy RV trabeculations. Left ventriculotomy can provide better exposure; however, it is associated with apical aneurysms, and with ventricular dysfunction sometimes necessitating heart transplantation.[5–12] In addition, the rate of residual defects is significant after surgical repair with a reoperation rate as high as 10% in infants.[5,11] This is even more significant when the defects are multiple, as in Swiss cheese mVSDs. Hence, device closure of mVSDs has become a valuable alternative to surgical patch closure, with encouraging results, particularly with the percutaneous transcatheter approach.[13,14] However, percutaneous closure requires the use of large venous sheaths, which may be associated with significant risk of vascular injury in childern. In addition, it remains a highly challenging procedure in infants. Thus, perventricular closure of mVSDs with a device deployed intraoperatively has emerged as an alternative approach in these cases. The same concept has been applied successfully for peratrial closure of secundum ASDs in small infants.[15]

In this article, we review the technique of device closure of mVSDs using the perventricular or hybrid approach off-pump without cardiopulmonary bypass (CPB). We discuss the indications, the selection criteria, the equipment required, and the potential complications encountered.

### Patient selection

Patients with significant mVSDs, who meet the criteria for closure of their defects, are considered for the perventricular or hybrid approach in the following conditions: (i) patients with contraindications for percutaneous device closure, which include small infants (<5.0 kg), in whom using large sheaths would be associated with significant morbidity, and patients with poor vascular access; (ii) patients with mVSDs and other cardiac anomalies requiring concomitant surgical repair (DORV, TGA); (iii) patients with multiple or Swiss cheese VSDs in whom surgical repair would provide suboptimal results and in whom percutaneous closure is highly challenging; (iv) patients with multiple mVSDs who had previously undergone pulmonary artery (PA) banding until they gain enough weight to have their VSDs closed.

### Pre-procedure evaluation

Prior to the procedure, these patients should undergo detailed echocardiographic assessment of their defects. This provides essential information regarding the number, location, and size of the defects as well as any associated cardiac lesions. The initial assessment is by transthoracic echocardiography (TTE), where the four-chamber and parasternal long-axis and short-axis views provide adequate planes to determine the location of mVSDs. In particular, the parasternal short-axis view near the tip of the mitral valve well delineates the location of these defects: inlet defects appear between 7 and 9 o'clock, mid-muscular defects between 9 and 12 o'clock, and anterior defects between 12 and 1 o'clock.

Additional imaging is usually performed intraoperatively by transesophageal echocardiography (TEE) to further plan and guide the procedure as detailed below. In some patients, the use of real-time 3-D echocardiography or cardiac CT imaging of the muscular septum, as part of the pre-evaluation procedure, can help demonstrate the exact morphology, size, and location of mVSDs.[16] The application of these imaging modalities is not routine, but may be helpful if additional views are required in more complicated defects, such as Swiss cheese VSDs, in which they could be superior to TTE or TEE.

### VSD devices

**Figure 1:**
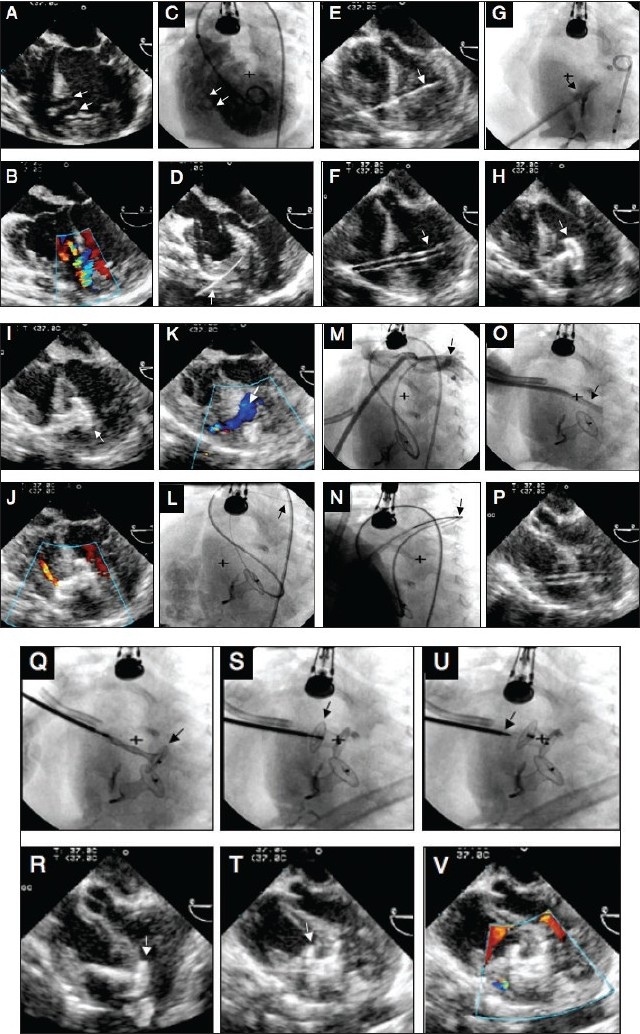
Transesophageal and fluoroscopic images in a 4-month old infant, 5.7 kg child with multiple muscular ventricular septal defects during perventricular device closure of the defects in the hybrid suite. A,B, TEE images without and with color Doppler in 4-chamber view demonstrating the two defects (arrows); C, left ventricular angiogram in the hepatoclavicular projection demonstrating the VSDs (arrows); D, TEE image during passage of the puncture needle (arrow) through the right ventricular free wall; E,F, images during passage of the guide wire and delivery sheath respectively into the LV cavity (arrow); G, angiogram using the sheath showing the tip of the sheath (arrow) in the left ventricle; H, deployment of the disk of the 12-10 mm Amplatzer Duct Occlud (arrow) in the LV cavity; I, deployment of the remainder of the Duct Occlud (arrow); J,K, images after the Duct Occlud has been released, both showing residual shunt (arrow); L, Cine fluoroscopy image during crossing of the residual defect from LV to the left pulmonary artery (arrow) (note the Duct Occlud is in good position); M, hand injection of the sheath that was positioned from the RV free wall to the main pulmonary artery; N, snaring of the wire from the left pulmonary artery (arrow); O,P, delivery sheath is well positioned in the LV cavity by fluoroscopy and TEE (arrow); Q,R, deployment of the LV disk of an 8 mm Amplatzer Muscular VSD device in the left ventricle (arrow); S,T, deployment of the right ventricular disk in the RV (arrow); U, release of the device from the cable (arrow); V, final color Doppler demonstrating no residual shunt.

Since the initial attempt of mVSD closure in 1988 with a double umbrella device,[17] the success of closure techniques helped support the innovation of newer devices that provide better closure results and better safety profile. Among these devices, the ones that have been used for perventricular mVSD closure include the modified Rashkind double umbrella device [no longer in use], the Clamshell Septal Occluder (C.R. Bard, Inc, Billerica, MA, USA) [no longer in use], the Cardio-SEAL device (NMT, Boston, MA, USA), and the Amplatzer Muscular VSD Occluder (AGA Medical, Plymouth, MN, USA). Unlike the first three devices, the Amplatzer Muscular VSD Occluder was designed specifically for the muscular septum. It was initially reported by Amin *et al*. in 1998 and first used in humans by Thanapoulos *et al*. in 1999.[18,19] It is made of 0.004-0.005” Nitinol wire with polyester mesh. The self-expandable discs are connected via a central waist, the diameter of which determines the size of the device. The waist is 7 mm long, and the two discs are 4 mm larger than the connecting waist. The device is available in sizes ranging from 4 to 18 mm in 2 mm increments, and it requires 6–9 Fr delivery sheath depending on its size. This device gained FDA approval in September 2007 for use in patients at high risk for surgical closure. It has several advantages that make it ideal for use in children. These include easy retrieval, simple delivery mechanism, and a small delivery system for deployment. These are mainly helpful for the transcatheter technique, but advantageous for the perventricular approach as well. In addition to these devices, the Amplatzer Duct Occluder has been used for closing mVSDs using the perventricular approach [Figure 1].[20] This mushroom-shaped device may be advantageous in cases where the RV muscle bundles, especially at the apex, prevent the expansion of the RV disk of the Amplatzer Muscular VSD Occluder.

### Equipment needed and closure protocol

Table 1 summarizes the essential equipment needed to perform the perventricular procedure. This is usually performed in the operating room under TEE guidance. In the event of complex anatomy with multiple or Swiss cheese mVSDs, the procedure can be performed in the catheterization laboratory, which allows the additional use of fluoroscopy (if the operating room is not equipped with fluoroscopy) to help guide crossing additional VSDs [Figure 1]. If the VSD is the only lesion, the procedure is performed without CPB. In case additional surgical intervention is needed to repair associated cardiac lesions, then CPB cannulas are inserted, but bypass is not initiated until after closure of the VSD. The heart is approached via a median sternotomy or a subxiphoid minimally invasive incision without sternotomy. Detailed evaluation of the defect, atrioventricular valves, and any associated anomalies is carried out by TEE. The atrioventricular valves are interrogated for baseline regurgitation, and distances from the aortic and tricuspid valves are measured to determine adequacy for device closure. The VSD is measured in different planes including the frontal four-chamber [Figure 1A,B] and basal short axis views. This helps in the selection of the device, which is usually chosen to be 1-2 mm larger than the VSD as determined by TEE (maximal size at end-diastole). If the procedure is performed in the catheterization laboratory, the VSD can also be delineated angiographically [Figure 1C],

**Table 1:** Equipment needed for perventricular closure of muscular ventricular septal defects

Purse string suture material
18-gauge needle
0.035″ short guide wire (Cook Inc., Bloomington, IN, USA); Terumo glide wire, regular length and exchange length
Introducer sheaths with dilators (7-10 Fr, 8-13 cm long)
Amplatzer muscular VSD devices in various sizes
Amplatzer duct occlud devices in various sizes
Amplatzer delivery system (cable/pin vise, loader) in various sizes
Gooseneck snares, 10 mm, 15 mm, 25 mm

After full evaluation, the surgeon and the echocardiographer need to determine the best location for puncturing the RV free wall. This is done by the surgeon by tapping the fingers or a hemostat on the RV free wall under TEE guidance. The location should be chosen away from the papillary muscles and moderator band, but perpendicular to the septum [Figure 1D]. The surgeon then places a 5.0 polypropylene purse-string at the chosen location. An 18-G needle (Cook Inc, Bloomington, IN, USA) is then introduced by puncturing the RV free wall [Figure 1D]. A 0.035” J-short guide wire (TSCF-35-80-3-BH; Cook Inc.) is passed through the needle and manipulated into the LV cavity across the mVSD [Figure 1E]. The needle is removed whilst keeping the wire positioned in the LV. A 7-10 Fr short introducer sheath (8-13 cm) is then passed over the wire with its dilator and advanced into the LV cavity [Figure 1F,G]. With the help of continuous TEE monitoring, it is essential to make sure not to advance the dilator too deep into the LV. This is because it could perforate the LV free wall despite the guidewire being in place. The dilator is then removed and the sheath tip kept in the LV mid-cavity. Sheath position can be checked by TEE and/or fluoroscopy and angiography [Figure 1F,G] if the procedure is performed in the catheterization laboratory. An appropriate-size VSD device is chosen and loaded onto the delivery cable under water or blood seal to prevent air bubbles. The device is advanced inside the delivery sheath under TEE and/or fluoroscopy guidance, until it reaches the tip of the sheath. The LV disk is then deployed in the LV mid-cavity by gentle retraction of the sheath over the cable. The whole cable/delivery sheath assembly is pulled toward the ventricular septum. The sheath is further retracted off the cable to deploy the connecting waist and then the RV disk. Continuous TEE monitoring is of extreme importance to confirm device position. If this is satisfactory, the device is released by counterclockwise rotation of the cable using the pin vise. A complete TEE study is performed to confirm device placement, assess for any residual shunting, and evaluate valve regurgitation that might have been induced by the device. The same procedure can be repeated in case of multiple mVSDs [Figure 1]. If the patient has other associated cardiac malformations requiring surgical repair, CPB is then initiated and surgical repair is performed. Otherwise, if the mVSD is the only lesion, the chest is closed in the usual fashion. Figure 1 demonstrates the various steps of perventricular closure of multiple VSDs. In this patient, the Amplatzer PDA device was used to close the apical VSD, and the muscular VSD device to close the other defect.

In patients with multiple mVSDs without additional cardiac lesions requiring repair, it is sometimes advantageous to perform the procedure in the catheterization laboratory as, on occasions, it is difficult to cross other defects after deploying the first device. In such cases, combining steps from both percutaneous and perventricular techniques may be particularly helpful to close the remaining VSDs [Figure 1]. Percutaneous access is obtained and additional VSDs can be crossed percutaneously under fluoroscopic guidance from the LV side [Figure 1L]. A wire is advanced into the pulmonary artery [Figure 1L]. Then, the surgeon places a short sheath in the pulmonary artery from the RV free wall [Figure 1M]. Under fluoroscopic guidance, a snare (Amplatz snare, ev3, Plymouth, MN, USA) is used to snare the wire from the pulmonary artery and exteriorize it out of the RV free wall [Figure 1N]. Once this loop is established, a short delivery sheath is advanced over the wire to the mid-LV cavity [Figure 1O,P]. The remaining steps are then carried out as in the routine perventricular approach described earlier for a single defect. This is a true hybrid approach since both percutaneous and perventricular techniques are used.[20]

### Advantages and disadvantages

The main advantages of this technique are avoiding CPB in the absence of associated cardiac lesions, and reducing CPB time in patients with additional cardiac anomalies requiring surgical repair. This is particularly important in patients who had or might need long cross-clamp time for repair of associated lesions or those who already have evidence of myocardial dysfunction. The procedure only requires a minimal incision in patients without additional lesions. Other advantages include avoidance of any ventricular incisions and avoidance of transection of RV muscle bundles. Unlike the percutaneous approach, it is not limited by weight or need for vascular access: mVSDs were closed in infants weighing as less as 3.2 kg using this approach.[29] It also avoids possible complications of rhythm disturbances and injury to cardiac valves from passing wires and large sheaths in repeated percutaneous techniques. The procedure is also relatively short with the time needed to cross the mVSD and deploy the device estimated in some studies to be less than 20 min, which compares very favorably to the percutaneous approach.[26] In addition, in cases of unusual orientation of the muscular septum as in DORV and TGA, it provides a much easier approach at crossing the VSD than the percutaneous technique by avoiding potential kinking of wires and sheaths.

In particular, this technique provides a major advantage to patients who present in the neonatal period for repair of associated cardiac lesions, such as newborns with coarctation of the aorta with significant mVSDs. This allows total repair in one setting for these patients. Using this technique, the indication for pulmonary artery banding for patients with multiple mVSDs would also be significantly decreased, and patients who had PA banding can have it debanded and the mVSDs closed intraoperatively.

The technique allows the immediate confirmation of adequate closure since it is done under TEE guidance, and any additional mVSDs can be easily detected and closed in the same sitting.

## RESULTS

Initial attempts at intraoperative device closure of mVSDs in patients, in whom percutaneous closure was contraindicated (e.g., small infants), had unsatisfactory results with mortality and failure rates as high as 14-25% and 20-40%, respectively.[21–24] These attempts consisted of an intraoperative approach involving device placement under direct vision via a right atriotomy across the tricuspid valve, after CPB and cardioplegic arrest were initiated.

The first successful case of intraoperative perventricular mVSD device closure without CPB on a beating heart was reported in a baby in 1998 by Amin *et al*.[25] Few series involving this technique then documented its efficacy and safety.[26–29] Table 2 summarizes the clinical data of some of the earlier studies performed on CPB as well as those done off-pump.

**Table 2:** Clinical studies involving perventricular closure of mVSDs

Study (reference^#^)	Number of patients	Age range (mean)	Weight (mean)(kg)	CPB for VSD device closure	Device used	Complications
Chaturvedi *et al*.[24]	4	0.5-29 years (8.75 years)	4.6-74 (26.0)	Yes	Modified Rashkind	One death non-related to procedure.
Murzi *et al*.[23]	5	4-41 months (14.4 months)	4.7-13.0 (7.96)	Yes	Rashkind device	One late death
Okubo *et al*.[21]	14	0.7-14 months (7.9 months)	3.0-11.0 (5.52)	Yes	Rashkind, Clamshell septal occluder, cardioseal	Two deaths, one patient needed transplant for dysfunction two patients needed PA banding
Lim *et al*.[28]	7	NA	3.0-7.0 (NA)	Not mentioned	Cardioseal	Failed procedure in one patient due to inability to expand RV disk; one patient with device malpositioned resulting in significant ventricular dysfunction
Amin *et al*.[25]	1	8 months	NA	No	Amplatzer muscular VSD occluder	None
Bacha *et al*.[27]	13	17 days-3 years (16.7 months)	3.0-20.0 (8.1)	No	Amplatzer muscular VSD occluder	One failed procedure in 13th patient due to inability to open RV disk
Diab *et al*.[29]	8	3 days-4.9 months (2.3 months)	3.2-7.3 (4.4)	No	Amplatzer muscular VSD and duct occluder	One patient with mediastinitis
Mercer-Rosa *et al*.[16]	1	1.5 months	3.5	No	Amplatzer muscular VSD occluder	Device embolization into LV

### Potential complications and how to avoid them

As mentioned above, detailed echocardiographic (TTE and TEE) evaluation of the defect and associated structures is a major step in planning the perventricular closure. Once planned appropriately, the procedure itself is significantly less complex than the percutaneous approach, as it involves fewer steps and less extensive manipulation of wires, catheters, and sheaths. To date, no significant major complications have been reported in the clinical studies involving the perventricular approach, when applied off-pump without CPB, despite the limited number of these studies. The interventionalist should, however, be meticulous in handling the equipment to avoid potential complications.

Two major potential complications are cardiac perforation and device embolization. Perforation is a rare but serious complication of the percutaneous technique and could occur with the perventricular approach, although it has not been reported to date. It may occur when introducing the delivery sheath and its dilator across the VSD into the LV as the dilator is stiff enough to puncture the LV if pushed too far. This is preventable by making sure the delivery sheath is positioned in the LV cavity not too close to the LV free wall by monitoring its position with TEE.

Device embolization is another major potential complication that could occur if the device is released prematurely or in an inappropriate position. The device can then embolize to the LV, ascending aorta, RV or pulmonary arteries. This has been reported in the literature in one case, in which the mVSD size was underestimated by TEE, resulting in the use of a small device that embolized into the LV cavity.[16] This may be preventable by using the appropriately sized device and by evaluating the device position by TEE prior to device release. If such a complication occurs, the surgeon can proceed with CPB and surgically close the VSD after removing the device.

Another potential challenge that might result in unsuccessful closure is the inability to deploy the RV disk of the device, when there is an apical mVSD and there are heavy RV trabeculations.[20,27,28] This problem may be avoided by using the mushroom-shaped Amplatzer Duct Occluder device [Figure 1I]. Thus, it is helpful to have these occluder devices available when planning perventricular closure, especially for apical mVSDs.

Valvular regurgitation can be a serious complication if the device impinges on the valve apparatus. This may potentially occur in some challenging mVSDs, such as high posterior mVSDs close to the atrioventricular valves. Thus, it is essential to measure the distance from the defect to the various valvar structures in order to select the appropriate device size and to monitor valve function by TEE prior to device release. Minimal valve regurgitation that is not hemodynamically significant can be followed up medically; however, if the device causes significant impingement on the valve apparatus after release, it should be removed surgically.

Hemolysis has been rarely reported with percutaneous device closure of mVSDs and could potentially occur with the perventricular technique, as it results from residual shunting.[30] By using the appropriate device size and avoiding under sizing, this complication should be avoidable. If hemolysis is significant, the residual shunt should be closed by implanting another additional device if possible, or the device should be removed and the defect closed surgically.

Air embolism is another serious complication. As in the percutaneous approach, the device should be loaded onto the delivery cable under water or blood seal to prevent such rare complications.

Conduction disturbances can be seen after mVSD device closure. Both right bundle branch block and complete heart block have been reported after percutaneous mVSD device closure,[30] although none has been reported with the perventricular approach so far. In addition, the perventricular approach avoids those rhythm disturbances associated with catheter manipulation seen with the percutaneous approach.

## CONCLUSION

Perventricular device closure of mVSDs is effective, safe, and provides a true hybrid approach to a challenging lesion by combining interventional and surgical techniques. Further experience with this technique will probably make it the approach of choice for small infants with significant mVSDs, patients with associated cardiac lesions undergoing surgical repair, and those with poor vascular access. Refinements of this technique might even expand its application to closing paramembranous VSDs in the future.
